# Cracking the Codes behind Cancer Cells’ Immune Evasion

**DOI:** 10.3390/ijms25168899

**Published:** 2024-08-15

**Authors:** Nikita Mundhara, Pritam Sadhukhan

**Affiliations:** Department of Oncology, Johns Hopkins University, Baltimore, MD 21287, USA

**Keywords:** cancer biology, cancer immunology, immune evasion, immunotherapy, targeted therapy

## Abstract

Immune evasion is a key phenomenon in understanding tumor recurrence, metastasis, and other critical steps in tumor progression. The tumor microenvironment (TME) is in constant flux due to the tumor’s ability to release signals that affect it, while immune cells within it can impact cancer cell behavior. Cancer cells undergo several changes, which can change the enrichment of different immune cells and modulate the activity of existing immune cells in the tumor microenvironment. Cancer cells can evade immune surveillance by downregulating antigen presentation or expressing immune checkpoint molecules. High levels of tumor-infiltrating lymphocytes (TILs) correlate with better outcomes, and robust immune responses can control tumor growth. On the contrary, increased enrichment of Tregs, myeloid-derived suppressor cells, and M2-like anti-inflammatory macrophages can hinder effective immune surveillance and predict poor prognosis. Overall, understanding these immune evasion mechanisms guides therapeutic strategies. Researchers aim to modulate the TME to enhance immune surveillance and improve patient outcomes. In this review article, we strive to summarize the composition of the tumor immune microenvironment, factors affecting the tumor immune microenvironment (TIME), and different therapeutic modalities targeting the immune cells. This review is a first-hand reference to understand the basics of immune surveillance and immune evasion.

## 1. Introduction

Throughout history, cancer has always been a matter of concern to the common people as well as to researchers and clinicians. Irrespective of sex, it affects millions of people each year, and the number is continuously increasing. In the present society, the significant factors behind cancer incidence remain to be the aging population, inappropriate lifestyle, imbalanced food habits, improved diagnosis, and greater public awareness [1,2,3,4]. From the onset of the disease to its extreme, the endogenous immune system plays a critical role in its progression. The immune system can fight back and eliminate the disease. The immune system constantly monitors the body for abnormal and cancer cells. Natural killer (NK) cells, cytotoxic T cells, and other immune cells recognize and eliminate these aberrant cells. However, recent research from the past couple of decades indicated that cancer cells or the tumor microenvironment can modulate the immune cells in the microenvironment [5,6,7]. Cancer cells can alter immune cell infiltration, differentiation, and function. Cancer cells rewire the immune network in a sequential and programmed manner where the immune system interacts with tumors, shaping their fate. Tumor cells may reduce the expression of surface antigens, making them less visible to immune cells. Tumors can release factors (e.g., TGF-β, IL-10, CXCLs) that suppress immune responses. Tumor cells express immune checkpoints such as PD-L1 and CTLA4, which bind on T cells, inhibiting their activity.

In some cases, immune cells such as TILs and macrophages infiltrate tumors. TILs include T cells, and B cells. Their presence can influence prognosis and response to therapy. Researchers have also identified ways to fight back to combat cancer cells’ strategies to escape the immune system [8,9,10,11]. Cancer treatments that target immune cells are collectively termed “**Cancer immunotherapy**”. Immunotherapies using checkpoint inhibitor drugs (e.g., anti-PD-1, anti-PD-L1, and anti-CTLA-4) enhance immune responses against tumors. They block inhibitory signals, allowing T cells to attack cancer cells effectively. Strategies such as adoptive T cell therapy and cancer vaccines aim to boost the immune response against tumors [12,13,14,15]. Despite success in clinical research, the apex is still not visible. Since cancer and associated pathophysiological conditions are a very dynamic process and unique in specific ways for each patient, a lot about the crosstalk between cancer cells and immune cells remains to be elucidated. Understanding the intricate interplay between tumors and the immune system is crucial for developing effective cancer treatments, and here in this review article, we aim to summarize various factors that can potentially affect cancer development and immune evasion and summarize the signaling crosstalk between cell types in the tumor immune microenvironment.

## 2. Composition of Immune Cells in the Tumor Microenvironment

Immune cells are heterogeneously infiltrated within the TME, which varies further with cancer type. This distribution, composition, and activation status of immune cells shape cancer progression and modulate therapy response. Rapid advancements in spatial transcriptomics and proteomics profiling allow dissecting the heterogenous immune landscape of solid tumors while retaining geographical information about the cellular architecture and functional phenotype of tumor, immune, and stromal cells [16,17]. This high-resolution information of TME enables clinicians to tailor precise treatments based on individualized immune profiles.

**Figure 1:**
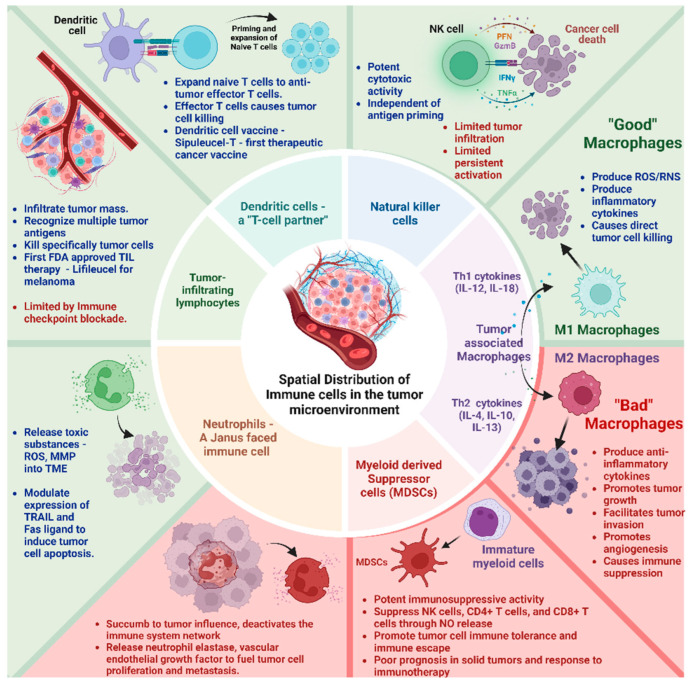
Spatial Distribution and Composition of Immune cells in the tumor microenvironment. Tumor-associated M1 macrophage, NK cells, Dendritic cells, and tumor-infiltrating lymphocytes have antitumor activity (Green box). Tumor-associated M2 macrophage and myeloid-derived suppressor cells have pro-tumor activity (Red box). Neutrophils have both pro and antitumor activity.

The immune landscape in the TME is composed of macrophages, neutrophils, natural killer (NK) cells, dendritic cells (DCs), bone marrow-derived suppressor cells (MSDCs), and tumor-infiltrating lymphocytes (TILs) (Figure 1). These cell types differentially interact with the cellular and molecular components of the TME engineering, either tumor progression or suppression, based on their activation status and interaction.

**i Tumor-associated macrophages (TAMs)-TAMs** are the most abundant **double-edged sword** immune cell type in the TME. They exert a multifaceted plasticity adapting to different polarization states dictated by the TME **M1 macrophages—“good macrophages,”** induced by Th1 cytokines (e.g., IL-12, IL-18) or activated Toll-like receptors produce reactive oxygen/nitrogen species (ROS/RNS) and pro-inflammatory cytokines (e.g., IL-1β, IL-6, TNF-α) causing direct tumor cell killing. **M2 macrophages—“bad macrophages,”** induced by Th2 cytokines (e.g., IL-4, IL-10, IL-13), produce anti-inflammatory cytokines (e.g., IL-10, IL-13, TGF-β) promoting tumor growth, ECM remodeling to facilitate tumor invasion, metastasis, angiogenesis, and immune suppression [18,19].

**ii Neutrophils—A “Janus–faced”** immune cell type. The circulating neutrophils and tumor-infiltrating neutrophils vary in their transcriptional signature and surface markers [20,21]. They release toxic substances, including reactive oxygen species (ROS) and matrix metalloproteinase 9 (MMP-9) within the TME [20]. Further, they modulate the expression of tumor necrosis factor-related apoptosis-inducing ligand (TRAIL) and Fas ligand, enhancing their capacity to induce tumor cell apoptosis. They synergistically activate other immune cells to bolster anticancer effects [20,21].

However, paradoxically, they can accumulate in solid tumors and succumb to tumor influence, triggering signaling cascades (e.g., JAK/STAT) that deactivate the immune system network. Their abundance correlates with poor prognosis and exhibits therapy resistance to immune checkpoint inhibitors [21,22]. In addition, their granular constituents (e.g., neutrophil elastase, vascular endothelial growth factor) fuel tumor cell proliferation, metastasis, and angiogenesis [23,24].

**iii Natural Killer (NK) cells**—These innate lymphocytes with potent cytotoxic activity can naturally recognize and kill tumor cells. Unlike T cells, NK cell activation is governed by interactions between NK receptors and target cells, independent of antigen processing and presentation [25,26]. Still, their effects on the solid tumor are limited. The main challenges involve inadequate tumor infiltration and persistent activation within the TME. Metabolic factors such as hypoxia, elevated adenosine, reactive oxygen species (ROS), and prostaglandins negatively affect NK cell activity [10]. Further, cancer-associated fibroblasts, tumor-associated macrophages, myeloid-derived suppressor cells, and regulatory T cells suppress NK cell-dependent anticancer immunity [27].

**iv Dendritic Cells (DCs)—a T-cell partner**. DCs infiltrate solid tumors, capture and process tumor antigens to transport them to the draining lymph nodes, and prime and expand naïve T cells into antitumor effector T cells. Activated DCs contribute to support effector functions during T-cell responses. They are associated with positive responses to immune checkpoint blockade (ICB) therapies. An inflamed solid tumor with T cells and DCs responds better to immunotherapies [28]. Further, based on the development pathway and lineage commitment, DCs have been classified as conventional or classic DCs (cDCs), which include cDC1s and cDC2s, plasmacytoid DCs (pDCs), and monocyte-derived DCs (moDCs). **cDC1s** specialize in cross-presenting intracellular antigens to CD8+ T cells via MHC class I molecules, playing a significant role in antitumor immunity. **cDC2s** present extracellular antigens to CD4+ T cells via MHC class II molecules, promoting various T helper cell responses (Th1, Th2, and Th17). **pDCs** are known for their ability to produce large amounts of type I interferons (IFN-α and IFN-β) in response to viral infections. They play a crucial role in antiviral immunity. They can also influence the adaptive immune response by activating T cells and promoting the differentiation of regulatory T cells12. **moDCs** are generated from monocytes, especially during inflammation or infection. They are potent antigen-presenting cells that activate CD4+ and CD8+ T cells. They induce strong antitumor immune responses and can be in vitro expanded from monocytes, making them a preferred choice for cancer immunotherapy [29,30,31].

**v Myeloid-derived suppressor cells (MDSCs)**—MDSCs originate from immature myeloid cells in the bone marrow and bear potent immunosuppressive activity. Their presence positively correlates with cancer stage, tumor load, poor prognosis in solid tumors, and response to immunotherapy [32]. Activated MDSCs suppress the antitumor immune response by inhibiting NK cells, CD4+ T cells, and CD8+ T cells through various mechanisms (e.g., nitric oxide release, depletion of essential metabolites, induction of other immunosuppressive cells, and expression of negative immune checkpoint molecules). They promote tumor cell immune tolerance and immune escape [33]. Some chemotherapies, such as decitabine and gemcitabine, can reduce circulating MDSCs in the system. All trans-retinoic acid (ATRA) can differentiate MDSCs to inhibit their activity. Ongoing research revolves around inducing apoptosis to selectively target MDSCs and improve responses to immunotherapy [34,35].

**vi Tumor-infiltrating lymphocytes (TILs)**–TILs–T (CD4+ and CD8+) and B lymphocytes infiltrate the tumor mass and recognize and destroy abnormal cells. Immune checkpoint inhibitors act as brakes to block their response. TILs come directly from the tumor and recognize many targets on cancer cells. This prevents the tumor from evading treatment by hiding one target at a time, offering a therapeutic advantage. TILs can be expanded in the lab to create a larger army of immune cells trained to attack the patient’s specific tumor [36]. Recent clinical trials have demonstrated good efficacy, tumor shrinkage, or complete response for TIL therapy in advanced solid tumors. In February 2024, the FDA approved the first cellular therapy for solid tumors called Lifileucel (Amtagvi). It utilizes TILs to treat melanoma, the deadliest form of skin cancer [37]. This therapy holds potential for other solid tumors as well, which collectively account for 90% of all cancer cases. Although TILs hold much promise, they can also become anergic, exhausted, and hypofunctional inside the TME. Reports indicate that lowering pH (6–6.5), like tumor mass, can render CD + T cells to an anergic state with impaired cytolytic and cytokine secretion activity. This indicates that TILs are probably in an anergic state in the TME [38]. Moreover, the TILs become dysfunctional and exhausted when inhibitory receptors such as PD-1, CTLA-4, and LAG3 are over-expressed on their surface. These receptors interact with their antigens on the tumor cells, leading to an inhibitory signaling cascade inside the TILs [39,40].

## 3. Cancer Cell Features That Regulate Immune Cell Function Activity

Delving into the intratumoral interaction between cancer and immune cells has long been a pivotal query in cancer biology [41,42,43]. The initial notion that immune cells could potentially regulate malignant transformation and progression was postulated as far back as 1909 by the esteemed German physician and scientist Dr. Paul Ehrlich [44]. However, it was not until nearly a century later that the concept of acquired cellular immunity’s role in carcinogenesis began to crystallize [45,46]. This journey of discovery, spanning the last three decades, has led to the development of the immunoediting concept [46,47]. The theory of immune editing elucidates the intricate functional manipulation of immune cells by cancer cells. In the tumor microenvironment, tumor cells and immune cells are in close contact and can affect the tumor progression in all the stages, such as premalignant transformation to metastasis [48]. The immune cells have a dual role as enhancers and blockers in cancer progression. This complex and orchestrated interaction between the cancer cells, immune cells, and cancer progression can be termed “cancer immunoediting”. The theory of cancer immunoediting solely stands on three distinct phases: Elimination, equilibrium, and escape [49,50,51]. Soon after the malignant transformation of cells, the endogenous innate and adaptive immune machinery come together to destroy the altered cells. This phase is called “Elimination”. If the process of Elimination reaches completion, the subject will be free of cancer, and the entire cascade will end. In sporadic events, a few altered cells manage to sustain the immune attack. Then, those altered cells will enter the next phase, “Equilibrium”. In this phase, the altered cells survive in a state of functional dormancy, and the cells emerge in an avatar where the endogenous adaptive immune machinery cannot recognize the cells and can induce an immunologically cold microenvironment. This final stage can be described as the “Escape phase”, and the state can be clinically diagnosed as a disease [49,50,52,53,54]. Here, a brief description of several extrinsic and intrinsic factors that can significantly regulate the function of immune cells and drive immune evasion is provided.

## 4. Extrinsic Factors Regulating the Immune Functions in the TME

Different extrinsic factors, from patients’ daily lifestyle to the tumor’s extracellular environment, significantly affect the composition of the immune cell types in the tumor tissue. They also affect the crosstalk between the tumor cells and immune cells and between different immune cell types. Individual patients’ age, race, sex, and hormonal regulation greatly affect the tumor immune landscape [55,56,57,58]. Obesity is another critical factor that regulates the premetastatic signals in specific immune cell types [59]. Interestingly, ICB-mediated therapy response is found to be more effective in obese patients compared to non-obese patients. Certain lifestyle features such as dietary habits, addiction to alcohol and nicotine, sedentary routines, and mental stress often can be correlated with the subduing antitumor immune responses [60,61,62]. Apart from these, different first-line anticancer therapies can potentially modulate the intra-tumor immune landscape. Reports suggest that cancer cells release several neoantigens after radiotherapy sessions in cancer patients [63,64,65,66].

Exposure to radiation increases the tumor cells’ immunogenicity by inducing interferon production and increasing the expression of MHC molecules. Overall, these mechanisms enhance the cytotoxic T-cell repertoire and ICB-mediated therapeutic response [63,66]. On the contrary, similar research studies also suggest that radiation therapy can induce the number of myeloid-derived suppressor cells (MDSCs) and immune-suppressive T cell repertoire. Tumor tissue in the human body is surrounded and connected to the adjacent organs through connective tissue, blood vessels, and different inflammatory cells. Altogether, these are called tumor stroma. The tumor stroma and the tumor tissue matrix are also shown to modulate the differentiation and function of the immune cells in the tumor microenvironment [67,68,69]. The lymphatic tissue around the tumor tissue can potentially act as a “gatekeeper” to reduce the infiltration of immune cells in the tumor tissue. The fibroblast, commonly termed cancer-associated fibroblast (CAF), is a specific cell type found in the tumor stroma that acts as a “Royal guard” to protect the tumor cells from the “Avengers” like immune cells. These CAFs can modulate the differentiation and function of different cytotoxic immune cells, such as T cells and macrophages. By secreting different immunosuppressive cytokines and chemokines, these CAFs promote the exhaustion of T cells and the re-polarization of antitumor M1-like macrophages into M2-like pro-tumorigenic macrophages [67,69,70,71,72].

## 5. Intrinsic Factors Regulating the Immune Function in the TME

**Figure 2:**
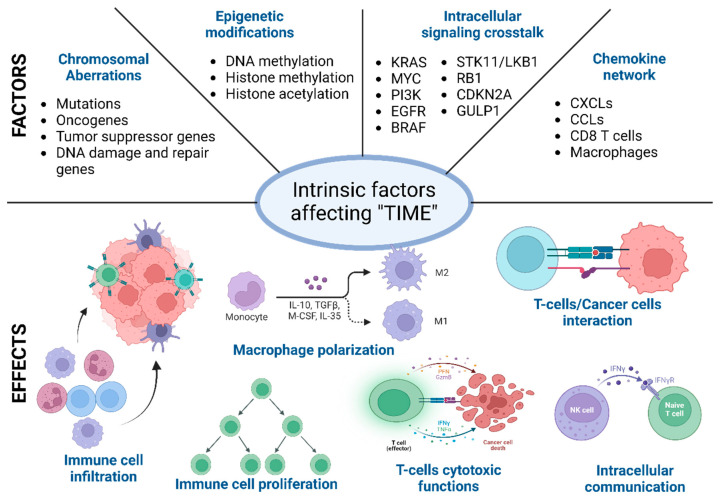
Different intrinsic factors affecting the tumor immune microenvironment (TIME). Schematic representation of intrinsic factors (primary and exclusive) that affect the immune cell recruitment, differentiation, and function in the TIME. These intrinsic factors often overlap, and overall, the immune microenvironment is regulated in a certain direction towards tumor elimination or promotion.

During the malignant transformation, the intracellular processes, starting from cell division to cellular metabolism, radicalize and run a “parallel government” within the host. These intrinsic functional and phenotypic changes in cancer cells are driven by chromosomal and epigenetic alteration, which leads to a differential gene expression pattern compared to self-cells [61,73]. These changes provide the cancer cells with a better environment to survive and expand. It is the first step towards immune evasion and onset of metastasis. Below, different factors behind these intracellular functional and phenotypic changes are described [74,75]. It is worth mentioning that these factors, such as the chromosomal and epigenetic alterations, differential gene expression, and changes in the secretome and metabolome, are often correlated and are often influenced by the external factors described above (Figure 2) [74,76].

### 5.1. Chromosomal Aberrations

Different chromosomal abnormalities, such as point mutations, insertions, deletions, amplification, or rearrangements, have been found to promote tumor initiation and progression in many cancer types [77,78,79]. Studies suggest that activation of different interferon genes in breast cancer cells induces the expression of an IL6-associated signaling cascade that promotes tumor progression [80]. On the other hand, high chromosomal instability predicts immunotherapy in many solid tumor types. Technological advancements in recent times, such as next-generation sequencing and single-cell sequencing, have allowed us to establish a correlation between the function of immune cell types and tumor mutation burden [16,17,73].

Genetic abnormalities in the DNA repair mechanism also increase mutational burden and chromosomal instability [81]. References suggest that mutation in the DNA mismatch repair system is correlated with enhanced infiltration of cytotoxic antitumor lymphocytes (CTLs) such as activated CD8+ T cells [82,83]. At the same time, it also upregulates the expression of different immune checkpoint proteins, such as PD-1, PD-L1, CTLA4, B7H3, LAG3, and indoleamine 2,3-dioxygenase [11,84]. Upregulated expression of these immune checkpoint regulators can mask the antitumor lymphocyte cytotoxic effect. Despite high infiltration of the CTLs, the tumor can efficiently evade the host immune system. High chromosomal instability increases cytosolic DNA, which activates the cGAS-STING pathway [85,86]. The cGAS-STING pathway can potentially inhibit immune escape by IFNβ1/CCL5-mediated infiltration of CTLs and can also promote tumor growth by activating VEGF signaling and immune checkpoints such as the PD-1 axis [85,87].

Mutation in different oncogenes such as KRAS, cMYC, EGFR, and HER can affect the immune cell activity in the tumor microenvironment (TME) through distinct mechanisms. Mutated KRAS suppresses the IRF2-mediated signaling and activates IL6 and IL8 to induce infiltration and differentiation of M2-like macrophages [88,89]. It also induces the enrichment of Treg cells through upregulated TGFβ and IL10. GM-CSF/CXCL3 signaling can potentially cause an immunologically cold microenvironment [90]. In MYC expressing TME, the CD47/PD-L1 axis goes up and inhibits the recruitment of cytotoxic immune cell types. Chromosomal aberration in tumor suppressor genes or signaling pathways like TP53, KRAS/STK11, and PTEN results in the upregulation of various cytokines and chemokines to accommodate the immunosuppressive immune cells in the TME. Mutated TP53 is often associated with NF-κB-driven IL8 and CXCL2 production to enrich immunosuppressive neutrophils [91,92].

### 5.2. Epigenetic Modifications

Similar to chromosomal abnormalities and instabilities, changes in the epigenetic landscape are also common in malignant transformation, tumor progression, and immune evasion. The epigenome in cancer cells can be modified through various cellular processes and can broadly be classified as DNA methylation, Histone methylation, and histone acetylation. All of the modifications in the epigenome have a dual role in altering intracellular communication and activity; these can be either tumor-supportive or tumor-suppressing [93,94].

DNA methylation is one of the critical factors responsible for normal growth and homeostasis. It can be of two types: hypo-methylation and hyper-methylation [95]. Hypomethylation is found in increased overexpression of immune checkpoints, the IL6/STAT3 pathway, VEGF signaling, and the upregulation of various chemokines [94,96,97]. Hypermethylation inhibits the expression of different HLA genes, resulting in decreased immunogenicity of the tumor cells. Hypermethylation is also seen in genes responsible for chronic and acute inflammation. Decreased immunogenicity is correlated with decreased recognition and recruitment of CD8+ T cells. Mutation in the IDH1/2 is significantly correlated with hypermethylation (reduced expression) of PD-L1, which can be tumor-suppressive [98,99]. However, mutated IDO1 signaling also correlates with suppressed CD8 + T cell activity through downregulated CXCL9/CXCL10 signaling. IDO1 mutation also upregulates G-CSF and correlates with non-suppressive neutrophil infiltration. This results in less inflammation (tumor-promoting) in the tumor microenvironment.

EZH2, EED, and SUZ12 comprise the PRC2 complex and are the primary regulators of histone methylation in cancer cells. Histone methylation typically modifies the Lys27 in H3 (H3K27me3). It suppresses the expression of different immunoregulatory genes regulating immunogenicity (MHC-I), macrophage, and MDSC activity (G-CSF, IL-6, CXCL9, and CXCL10). It can also decrease the enrichment of CD8+ T cells in the TME [100,101,102].

Histone acetylation is another main hallmark of epigenetic modifications in cancer cells. Through histone acetylation, immune responses are altered by the expression of histone acetyltranferase1 (HAT1) and histone deacetylases (HDACs). HAT1 upregulates the expression of PD-L1, while different HDACs can reverse the activity of HAT1. HATs and HDACs can also regulate the cytokine and chemokine network in the cancer cells to impair T cell activity in the TME [103,104,105].

### 5.3. Intracellular Signaling Crosstalk

In the last two decades, extensive research has identified several genes in cancer cells that potentially affect the function of different immune cell types. These gene types either increase or decrease the immunogenicity of the cancer cells or can also rewind the tumor immune microenvironment through different membrane proteins mediated signaling or secretory factors such as exosomes. Intracellular signaling in the cancer cells determines the immune reactivity of the tumor microenvironment [106,107]. The expression of specific oncogenes such as KRAS, MYC, PI3K, EGFR, and BRAF can downregulate the MHC genes such as MHC class I, MHC II, CD80, and CD86. The downregulation of the MHC genes can impair T cell recognition and activation. KRAS-G12D mutation results in an elevated level of IL6 secretion in the TME [89,108,109]. Increased levels of IL6 can be correlated with the enrichment of immunosuppressive cell types such as Tregs and M2-like macrophages. The enrichment of immunosuppressive cells in the TME leads to immunologically “cold” TME KRAS-G12D expression, which is also found to correlate with GM-CSF, which leads to increased accumulation of the myeloid-derived suppressor cells and less accumulation of cytotoxic CD8+ T cells [110]. Similarly, KRAS-G12D mutation also drives several other immunosuppressive pathways mediating IRF2, CXCL3, CXCL3, IL10, and TGFβ. Similar to KRAS, MYC is another oncogene that drives several immunosuppressive mechanisms by increasing the expression of CD47 and PD-L1. Therapeutic targeting of MYC reduces CD47 and PD-L1, which results in tumor regression [111,112]. The oncogenic molecule EGFR also has a similar immunosuppressive role through activating the CD73-mediating signaling cascade [113].

Apart from the oncogenes, several tumor suppressor genes are also involved in immune evasion. Silencing tumor suppressor genes such as TP53 and PTEN results in spontaneous tumor initiation and immune evasion [81]. The immunosuppressive pathways are driven through the expression of different oncogenic chemokines, such as CCL3, CCL11, CXCL5, and macrophage-stimulating factor (M-CSF). The absence of TP53 results in the expression of different Wnt ligands, which are critical in driving diverse pathways leading to immune evasion [114]. The mutation or loss of STK11/LKB1, RB1, TET2, GULP1, and CDKN2A107 expression also results in severe malignant conditions by altering the tumor immune microenvironment [81,115]. Table 1 depicts the key molecular pathways in critical cancer types.

### 5.4. Chemokine Network

Immune cells are the frontline defenders in the human body’s battle against malignant cells. In this life-or-death struggle, chemokines are crucial in guiding the immune cells. These small signaling proteins can stimulate immune cells to leave the bloodstream and enter the tumor tissue, where they can carry out their functions. Chemokines also influence the lifespan and differentiation of immune cells and can either promote or inhibit their antitumor activities. In essence, the chemokine network acts as a “conductor”, orchestrating the immune system’s response to the tumor tissue [116,117,118].

Tumor cells in tissue produce several chemokines, such as CXCL1, CXCL2, CXCL3, CXCL5, CXCL10, CXCL12, CCL7, CCL2, and CCL5, to facilitate their survival and expansion. Tumor cells also express different chemokine receptors like CXCR2, CXCR3, and CXCR4 to drive proliferative signaling cascades [116,117,118]. The overexpression of both chemokine secretion and chemokine receptors creates a feedback loop mechanism and promotes faster proliferation of cancer cells. Chemokines have a role in all stages of cancer progression. CXCL12 has been reported in many research articles as a potent inducer of angiogenesis. Other chemokines, such as CXCL1, CXCL2, and CXCL8, were also found to promote angiogenesis. On the contrary, chemokines such as CXCL4, CXCL9, and CXCL10 are found to inhibit angiogenesis. ELR+ (Glu-Leu-Arg) chemokines are found to be inducers, and ELR- are found to be inhibitors of angiogenesis. Like angiogenesis, these chemokines also have a potential role in promoting metastasis in many solid cancer types [116,119,120,121].

Chemokines are magically pleiotropic in nature. Among the vast pool of information regarding the effects of chemokines in tumor progression, it is noteworthy that CXCR6 is reported to enhance the activity of cytotoxic CD8+ T cells and natural killer T cells [122]. CCR5 expression on effector T cells improves their immunogenicity through the CD40/CD40L axis [123]. Puzzlingly, numerous reports are also available that certain chemokines such as CCL22, CCL7, and CCL28 can potentially be involved in the recruitment of specific subsets of immune cells that can suppress the antitumor effector immune cells [116,124]. These chemokines can upregulate tumor-associated M2-like macrophages and myeloid-derived suppressor cell recruitment [125].

## 6. Targeting the Immune System for Solid Cancer Therapy

Dr. William B. Coley, often regarded as the father of immunotherapy, laid the foundation for cancer immunotherapy. In the late 19th century, Dr. Coley’s “Coley’s toxins”, a mixture of heat-killed bacteria, including Streptococcus pyogenes and Serratia marcescens, remarkably caused tumor regression and improved survival in some of his patients. He hypothesized that inducing fever could stimulate the body’s natural immune system against cancer [126,127]. Cancer immunotherapy seeks to activate the host immune system and rewire immune cells to specifically target and destroy tumor cells, ultimately delivering long-lasting cancer-free survival [128]. Unfortunately, the TME frequently exhibits an imbalance between immune stimulatory and inhibitory pathways. The patient’s natural anticancer immunity dampens their response to immunotherapy treatments. The cytokines and chemokines in the TME engineer the pro- and anti-tumorigenic activities and thereby guide the MDSCs and Tregs to obstruct tumor immunity, while NKs, CD8+, CD4+ T cells, M1 macrophages, and DCs to stimulate an antitumor response [6,17]. Targeting the TME to restrain immunosuppressive pathways and enhance cellular immune function may improve responses in advanced solid tumor patients. One of the main obstacles in treating a cancer patient is tumor heterogeneity [129]. The concept of tumor heterogeneity, i.e., understanding the fact that each tumor is within a patient or among the patients, can vary to a huge extent in terms of intra/inter-cellular signaling and immune cell composition. It took shape like a “snowball”; the more the extent of cancer research deepens through the advancement of molecular biology and genetics, the more the magnitude and effect of tumor heterogeneity are understood. In recent times, studying and understanding tumor heterogeneity is one of the main areas of research and clinical focus. Tumor heterogeneity can be of many types. It can be spatial (between cells in different regions of a tumor) or temporal (the composition and signaling changes over time). It can also be genetic (different cells can harbor different mutations in the genome) or phenotypic (depending on the tumor microenvironment, cellular function and extracellular communication can be different) [130,131]. Here, we discuss the multiple tools researchers have investigated to target the TME.

### 6.1. Immune Checkpoint Inhibitors (ICIs) for the Treatment of Solid Cancers

Immune checkpoints prevent excessive immune response to stop killing healthy cells. Unfortunately, in the TME, these checkpoints, such as CTLA-4, programmed death receptor–1 (PD-1), Nectin-4, and lymphocyte activation gene 3 (LAG3) on T cells, are engaged with their partner ligand on tumor cells to downregulate immune response, leading to reduced cytotoxicity. ICIs sequester the checkpoint proteins and prevent their binding to cognate ligands, averting the inhibitory signaling cascade [11,84,132].

In 2011, the first FDA-approved I.C.I., Ipilimumab (Yervoy), targeted CTLA-4 for late-stage melanoma [133,134]. Shortly after, Nivolumab (Opdivo) and Pembrolizumab (Keytruda)—which targets PD-1—were approved in 2014 for advanced melanoma [135,136]. In 2015, Opdivo was approved to treat advanced lung cancer, metastatic RCC, and Keytruda for NSCLC [137,138,139]. The next year followed the approval of Opvido and Keytruda for first-line treatment of head and neck cancer [140,141]. In 2019, enfortumab vedotin-ejfv (Padcev)—which targets Nectin-4—was approved for metastatic urothelial cancer [142]. Subsequently, Opvido was approved for metastatic esophageal squamous cell carcinoma in 2022 [141] and combined with cisplatin and gemcitabine for metastatic urothelial carcinoma in 2024 [143]. Similarly, Keytruda has been approved in combination with chemotherapy for unresectable or metastatic biliary tract cancer, HER2 negative gastric or gastroesophageal junction adenocarcinoma, microsatellite instability-high or mismatch deficient solid tumors in 2023 [144], and for stage III-IV cervical cancer in combination with chemoradiotherapy in 2024 [145].

In recent years, combinatorial ICIs have been FDA-approved for multiple solid cancers. In 2020, Opvido, in combination with Yervoy, was approved as a first-line treatment for patients with metastatic NSCLC and unresectable malignant pleural mesothelioma [146,147]. Opdualag—a combination of nivolumab (targets PD-1) and relatlimab (targets LAG-3)—was approved for metastatic melanoma in 2022. This combinatorial therapy increased T-cell activation compared to the activity of either antibody alone. Padcev, in combination with Keytruda, was approved for advanced bladder cancer in 2023 [148].

### 6.2. Bispecific Antibodies for the Treatment of Solid Tumors

Bispecific antibodies (BsAbs) are genetically engineered recombinant antibodies that simultaneously target two independent antigens. This leads to a “double blockade” effect that can enhance anticancer responses compared to single-target antibodies. They bind to tumor-associated antigens (TAAs) expressed on tumor cells and immune cells, bridging the gap between the immune system and cancer cells. BsAbs can be engineered in various formats, including bispecific T-cell engagers (T-BsAbs), which engage CD3 and TAAs on tumor cells. The dual targeting property of BsAbs allows them to modulate immune responses, enhance T-cell activation, and promote tumor cell killing [149,150].

While BsAb drugs have been clinically effective and approved for hematologic malignancies, no BsAb has yet been approved for solid tumors. However, over 100 BsAbs targeting various solid malignancies have shown promising preclinical results and are currently being evaluated in clinical trials. These BsAbs target antigens such as EpCAM, C.E.A., PMSA, and ErbB family members. The suppressive tumor microenvironment (TME) in solid tumors poses challenges for BsAbs [151,152]. Immune deficiency within the TME can hinder T-cell activity. Strategies to overcome this include improving T-cell infiltration, enhancing BsAb efficacy, and addressing adverse effects. While no bispecific antibody has been licensed for solid tumors, ongoing research and clinical trials hold promise for their future application in treating these challenging cancers [153].

### 6.3. Oncolytic Viruses for the Treatment of Solid Tumors

**Oncolytic viruses** are designed to infect and selectively replicate within cancer cells, leading to their destruction while sparing healthy cells. They exploit the unique features of tumor cells, such as defective antiviral responses and increased expression of specific receptors. Once inside the tumor, these viruses replicate and lyse cancer cells, releasing more viral particles and destroying the tumor mass [154,155].

Several viruses, such as adenoviruses, have been investigated for their oncolytic properties, which can infect many cell types. Measles-based oncolytic viruses have demonstrated tumor-specific replication. Vaccinia and enteroviruses exhibit oncolytic properties. **T-VEC (Talimogene Laherparepvec)** is an HSV-based oncolytic virus approved for treating advanced melanoma. It directly injects the virus into melanoma lesions, leading to tumor regression [156,157]. **Rigvir** is a non-engineered oncolytic virus derived from the ECHO-7 virus. It has been used in Latvia for decades to treat various cancers [158]. **Pelareorep (Reolysin)** is a reovirus-based oncolytic therapy being investigated in clinical trials for multiple cancer types [159].

Clinical trials have evaluated oncolytic viruses as monotherapies and in combination with other treatments (e.g., chemotherapy, immunotherapy). Challenges include optimizing viral delivery, minimizing immune clearance, and addressing potential toxicity. Researchers continue to explore novel oncolytic viruses, improve their safety profiles, and enhance their tumor-targeting capabilities [155].

### 6.4. Cancer Vaccines for the Treatment of Solid Tumors

Cancer vaccines are either preventive or therapeutic. **Preventive vaccines** aim to reduce the risk of cancer development and must be administered before the infection. The HPV Vaccine protects against human papillomavirus (HPV), which is linked to cervical, anal, throat, and other cancers. The Hepatitis B vaccine reduces the risk of liver cancer associated with HBV. Preventive vaccines only protect against specific oncoviruses and cannot prevent cancers caused by other factors (genetics, environmental exposures). **Therapeutic cancer** vaccines treat existing cancers or prevent relapse/metastasis by activating the immune system to recognize and attack cancer cells [160,161]. However, therapeutic vaccines are challenged by the suppressive action of TME and the tolerance to tumor-associated antigens (TAMs). Cancer vaccines are of three significant categories [162,163].

**Cellular vaccines** are developed using autologous (patient’s own) tumor cells or allogeneic (from another person) tumor cell lines. In dendritic cell vaccines, patient-derived dendritic cells are loaded with tumor-associated antigens (TAAs) and reintroduced into the patient. These dendritic cells, as vaccines, work as antigen-presenting cells (APCs) to stimulate an immune response against the tumor by activating the CD4 and CD8 T cells [164]. **Sipuleucel-T (PROVENGE)** is an autologous cellular immunotherapy approved for metastatic castration-resistant prostate cancer (mCRPC) and administered to patients with minimal or no symptoms [165,166,167]. Sipuleucel-T is customized for each patient using their antigen-presenting cells (APCs). APCs are exposed to a fusion protein (PA2024) containing prostatic acid phosphatase (PAP) antigen. The activated APCs are then infused into the patient, stimulating an immune response against PAP-expressing prostate cancer cells. PROVENGE has shown a survival benefit in mCRPC patients [165,168].

**BCG (TheraCys & TICE):** BCG stands for Bacillus Calmette-Guérin, a live attenuated strain of Mycobacterium bovis. It is used primarily as a treatment for early-stage bladder cancer (non-muscle-invasive) [169,170]; however, it is currently investigated for its potential in other cancers and immunotherapy. It is directly instilled into the bladder after transurethral resection of bladder tumors (TURBT). BCG activates immune cells (macrophages, T cells) to attack cancer cells, leading to an inflammatory response, targeting residual tumor cells and preventing recurrence [169,170,171]. **GVAX vaccines** are created by genetically modifying tumor cells to express immune stimulatory factors such as granulocyte-macrophage colony-stimulating factor (GM-CSF) [172,173]. The modified tumor cells attract immune cells to the tumor site by secreting GM-CSF, promoting antitumor immunity. GVAX vaccines have been investigated in clinical trials with mixed results for various cancers, including pancreatic and prostate cancer [172,174,175].

**Viral Vector vaccines** are modified viruses as delivery vehicles to introduce tumor antigens into the body. These viruses are engineered to express specific tumor antigens. Once inside the body, they promote T-cell responses against the tumor, adenoviruses, and other viral vectors that deliver tumor-specific antigens [176,177]. **VAC85135** is an exciting cancer vaccine developed by Nouscom in collaboration with Janssen Research & Development, LLC (Janssen). These viruses carry genetic material encoding protein fragments expressed only in cancer cells to train the immune system to recognize and attack cancer cells expressing these abnormal proteins [178]. VAC85135 has advanced to the Investigational New Drug (IND) stage, signifying its potential for clinical use. **NOUS-PEV** is a vector-based vaccine and expresses 60 neoantigens, unique protein fragments found in cancer cells [179,180]. A phase Ib trial has been conducted to evaluate NOUS-PEV in combination with pembrolizumab (an anti-PD-1 checkpoint inhibitor) for patients with metastatic melanoma. This vaccine is based on a **heterologous GAd20/MVA prime-boost strategy.** The heterologous GAd20/MVA prime-boost strategy is an innovative approach to vaccine development. GAd20 is a replication-deficient adenovirus serotype 20 vector that delivers tumor-specific antigens (such as neoantigens) to antigen-presenting cells (APCs) in its prime phase. These APCs process and present the antigens to T cells, priming the immune system. During the boost phase, MVA (Modified Vaccinia virus Ankara) serves as a second vector to deliver the same tumor-specific antigens, reinforcing the immune response [179,180].

**Molecular vaccines** include mRNA-based vaccines, DNA vaccines, and peptide-based vaccines. mRNA vaccines for COVID-19 instruct cells to produce tumor antigens, eliciting an immune response. **mRNA-1345(mRESVIA)** is approved for the adjuvant treatment of patients with high-risk melanoma following complete resection in combination with KEYTRUDA [181]. mRNA-1345 encodes a stabilized prefusion F glycoprotein found on the RSV virus [182]. It instructs cells to produce this protein upon administration, stimulating an immune response [183,184]. **WGc-043 (EB Virus-Related mRNA Therapeutic Cancer Vaccine)** is the world’s first approval of an EB virus-related mRNA therapeutic cancer vaccine targeting EB virus-related cancers, including nasopharyngeal carcinoma (NPC) [185]. It also encodes a stabilized prefusion F glycoprotein like mRESVIA, found on the EBV virus, and demonstrates promising efficacy and low toxicity compared to other mRNA cancer vaccines. Ongoing clinical trials aim to evaluate its effectiveness in treating advanced EBV-positive solid tumors [185,186].

**DNA vaccines** introduce tumor DNA sequences to stimulate immunity. **INVAC-1**, an innovative DNA vaccine, targets the **human telomerase reverse transcriptase (hTERT)**, which is highly expressed in over 85% of human tumors and is designed to target advanced solid tumors [187]. It encodes a modified hTERT protein devoid of catalytic activity. The N-terminal part of hTERT, including the nucleolar localization sequence, is replaced by a ubiquitin moiety. This modification enhances peptide presentation and stimulates specific anti-hTERT CD4 and cytotoxic CD8 T-cell responses. It was safe, well-tolerated, and immunogenic in patients with relapsed or refractory cancers [187,188]. **DoriVac (DNA Origami Platform)** utilizes self-assembling DNA origami nanostructures [189]. DNA strands can be precisely engineered to fold into specific shapes (DNA origami scaffold) and strategically place the tumor-specific antigens (neoantigens) and immune-stimulating molecules (adjuvants) using complementary base pairing. The DNA origami acts as a carrier, presenting antigens and adjuvants to immune cells with precise spacing to enhance immune activation and minimize off-target effects. It has shown promise in preclinical studies against melanoma, colon cancer, lung cancer, and neuroblastoma. However, clinical use in humans is still under investigation [189].

**Peptide-based vaccines** contain specific tumor antigen peptides to activate T cells [190,191]. **Synthetic long peptides (SLPs)** are entirely synthetic, carefully selected peptide sequences, each 20 to 35 amino acids long. These peptides are longer than typical immunogenic epitopes and are crucial for effective immune system activation. SLPs are directly taken up and processed by antigen-presenting cells (such as dendritic cells) to elicit both CD4+ helper and CD8+ killer T cell responses. SLPs harbor multiple T cell epitope sequences, enhancing immune recognition and response [190,191,192]. **SVN53-67/M57-KLH (SurVaxM)** stimulates an immune response against survivin, a tumor-associated antigen (TSA) highly expressed in glioblastoma and other solid tumors [193]. It contains a synthetic long peptide (SLP) mimic of survivin, an immunogen. The SLP is combined with KLH (keyhole limpet hemocyanin), a carrier protein that enhances the immune response. Early results suggest safety and immunogenicity, but further studies are needed to assess its efficacy in improving patient outcomes [193,194]. IO Biotech’s innovative therapeutic cancer-vaccine technology, **T-win**, focuses on recruiting naturally occurring T cells to disrupt immunosuppressive processes within the tumor microenvironment (TME) [195]. T-win product candidates consist of peptides derived from TME-specific molecular target antigens. These peptides activate pre-existing CD4+ and CD8+ T cells in draining lymph nodes. The resulting T cells migrate to the TME, eliminating immunosuppressive target cells directly or releasing pro-inflammatory cytokines. This shift from an immunosuppressed to an inflamed TME enhances the antitumor response [195]. **IO102-IO103** is a dual-peptide vaccine targeting indoleamine 2,3-dehydrogenase (IDO) and programmed death ligand 1 (PD-L1). It nullifies two different immunosuppressive mechanisms in the TME. IO102-IO103 is in a phase 3 clinical trial for melanoma, combined with the PD-1 inhibitor pembrolizumab. It might exhibit potential in non-small-cell lung cancer (NSCLC) and squamous-cell carcinoma of the head and neck (SCCHN) [196,197].

### 6.5. Adoptive Cell Therapy for the Treatment of Solid Tumors

Adoptive cell therapy (ACT) for solid tumors encompasses various strategies to enhance the activity of T lymphocytes and other effector cells involved in the antitumor immune response. **Chimeric Antigen Receptor (CAR.) T cells** are genetically engineered T cells that express new chimeric antigen receptors to recognize specific tumor surface proteins. They have shown remarkable results in some hematologic malignancies and are currently under clinical trials for refractory solid tumors [198,199]. Unlike CAR–T cells, which target tumor cell surface proteins, **T-cell receptor (TCR) Gene-Modified T Cells** (TCR T cells) recognize peptides derived from proteins across all cellular compartments, expanding the horizon of potential targets. They are generated by introducing a TCR gene sequence specific to tumor-associated antigens presented in the context of specific HLA (usually class I) molecules [200]. **Tumor-infiltrating lymphocyte (TIL)** therapy involves infusing autologous TILs into patients. It has demonstrated safety and promising activity, particularly in advanced melanoma [201]. In 2024, the FDA approved the first cell-based therapy for solid tumors, specifically advanced melanoma. This groundbreaking therapy uses tumor-infiltrating lymphocytes (TILs) harvested from a patient’s tumor [202]. These naturally existing T cells are encouraged to multiply into billions of cancer-fighting cells in the laboratory and then reintroduced to the patient. The treatment, marketed as **Lifileucel** (commercially known as **Amtagvi**), has shown promising results, with approximately 30% of patients experiencing tumor shrinkage or disappearance [202]. While CAR-T therapies have been successful in blood cancers, this approval marks a significant step toward expanding cell-based therapies to solid tumors.

**Chimeric Antigen Receptor (CAR)–macrophage therapy** involves engineering macrophages to express chimeric antigen receptors that can specifically recognize and eliminate cancer cells, enhancing the immune response. Nanoparticles have emerged as promising translational tools to tackle tumor-promoting TAMs [203]. They can restrict TAM survival, inhibit TAM recruitment to tumors, and re-polarize tumor-supportive TAMs toward an antitumor phenotype. These nanoparticles mimic antigen-presenting cells (APCs) and deliver immune checkpoint inhibitors to the tumor sites [9,204]. Recent efforts are gearing towards developing **“Off-the-shelf” NK cell-based cancer immunotherapy**—ready-to-use, standardized natural killer (NK) cell products that can be administered across multiple patients without needing individualized cell processing. It can offer immediate availability, cost efficiency, and consistent quality. Several clinical trials are assessing the safety, efficiency, and long-term outcomes of such therapies [205,206]. More than 500 registered clinical trials of **DC-based immunotherapy** in solid cancer have proven its safety and reliability [29,30,31]. Many clinical trials for prostate cancer, hepatocellular carcinoma, and NSCLC have tested moDCs for immunotherapy, giving promising results. Some trials have also tested cDC and pDC for metastatic melanoma, providing mixed responses [31,207]. Further, **dendritic cell-derived exosomes (Dexs)** contain processed peptides derived from antigenic material, presenting them on their surface with MHC class I (MHC-I) and MHC class II (MHC-II), along with co-stimulatory molecules that can activate APCs against tumors. Multiple Dex-based clinic trials are ongoing to demonstrate its safety and feasibility [32].

## 7. Conclusions and Future Perspectives

The 150-year journey of cancer immunotherapy has evolved from Coley’s toxin to advanced cancer vaccines and CAR-T therapies. Emerging spatial technologies have contributed to understanding the diversity of immune cells lodging in the tumor microenvironment. Some immune cells either induce immune surveillance or are antitumor in nature. In contrast, some under the influence of tumors become pro-oncogenic and either promote tumor development or inhibit the activity of anti-tumor immune cells. Cancer immunotherapy involves several arms to either reduce the tumor load by directly eliciting an immune response or transform cold tumors into hot ones, enhancing their vulnerability to immunotherapy.

Cancer vaccines in the form of dendritic cells loaded with patient-derived neo-antigens (neo peptides formed by the tumor) trigger an immune response against cancer by activating the CD4 and CD8 T cells [30,163]. Checkpoint blockade therapies using checkpoint inhibitors or bispecific antibodies block the interaction between tumor cells and immune cells. This stops the inhibitory action of tumor cells toward the immune cells [11,84,132]. Further, antibody-drug conjugates also deliver the drug to the tumor site alongside its blockade activity [208,209,210]. Currently, novel techniques such as PROTACS (Proteolysis-targeting chimeras) are being explored to downregulate the expression of checkpoint inhibitors. PROTACs are molecules that exploit the ubiquitin-proteasome system (UPS) for targeted protein degradation [211]. PROTACs have been designed to degrade PD-1 and PD-L1 proteins [212].

Cancer immunotherapy trains the immune system to recognize and attack cancer cells and offers a viable solution to cancer reoccurrence. It creates an immune memory effect, allowing the patients to live cancer-free for longer with fewer side effects. Although immunotherapy works for less than half of patients with partial responses, combining it with existing treatments (such as chemotherapy or radiotherapy) can improve their effectiveness in the near future.

## Figures and Tables

**Table 1:** The role of key tumor-intrinsic signaling pathways in critical cancer types.

Signaling Pathway	Tumor Type	Effects
PI3K/Akt/mTOR Pathway	Breast Cancer, Prostate Cancer	Promotes cell growth and survival
		Promotes tumor progression
		Develop therapeutic resistance
		Induces the expression of immune checkpoints e.g., PD-L1, CTLA-4
		Downregulate MHC expression
		Enhance the recruitment of immunosuppressive cells like Tregs and myeloid-derived suppressor cells (MDSCs).
MAPK/ERK Pathway	Melanoma, Non-small-cell lung carcinoma	Induces the expression of immune checkpoints, e.g., PD-L1, CTLA-4
		Downregulate MHC expression
		Influence macrophage polarization from M1 to M2 phenotype
		Affect T cell activation and function
		Upregulate immunosuppressive cytokines like IL-10 and TGF-β
		Develop resistance to immunotherapy
Wnt/β-Catenin Pathway	Colorectal Cancer, Hepatocellular Cancer	Promote differentiation and activity of Tregs
		Influence macrophage polarization from M1 to M2 phenotype
		Downregulate antigen presentation pathway
		Affects the cancer-associated fibroblast and extracellular matrix, promoting tumorigenesis
		Promotes EMT pathways
		Develop resistance against adoptive T cell therapies
NOTCH Signaling Pathway	Leukemias, Breast Cancer	Regulates T cell function
		Promote the development of exhausted T cells
		Promote the M2 macrophage phenotype
		Induces PD-L1 expression
		Influence MDSC infiltration
		Promotes EMT pathways
Hedgehog Signaling pathway	Basal Cell Carcinoma (BCC), Medulloblastoma	Influence tumor cell proliferation and survival
		Influence the behavior of stromal cells
		Develop reduced immunogenicity and immunotherapy resistance
		Involved in the production of prostaglandins
		Regulates immune-suppressive cytokine production through its transcription factors, such as Gli1, Gli2, and Gli3
		Elevate IL-6 levels in the tumor microenvironment
Jak/STAT Pathway	Hematological Malignancies, Solid tumors	Regulate Immune Cell Function
		Impact immunosuppressive Cytokine Production
		Impair the activation of CD4+ T helper cells
		Upregulates the expression of CXCR2-associated ligands
P53 Pathway	Ovarian Cancer, Lung Cancer	Promotes anti-inflammatory cytokines
		Induce genomic instability and a higher mutation rate.
		Develop therapeutic resistance
		Upregulates CCL2 and CXCR4, promoting MDSC infiltration
		Facilitates the expansion and activity of Tregs
		Influence extracellular matrix (ECM) remodeling through cytokines and chemokines.
		Impair TNFα production and function
